# Is It Time to Rethink Our Weight Loss Paradigms?

**DOI:** 10.3390/biology9040070

**Published:** 2020-04-02

**Authors:** Paulo Gentil, Ricardo Borges Viana, João Pedro Naves, Fabrício Boscolo Del Vecchio, Victor Coswig, Jeremy Loenneke, Claudio André Barbosa de Lira

**Affiliations:** 1 College of Physical Education and Dance, Federal University of Goiás, Goiânia 74690-900, Brazil; vianaricardoborges@hotmail.com (R.B.V.); jpanaves12@gmail.com (J.P.N.); andre.claudio@gmail.com (C.A.B.d.L.); 2 School of Physical Education, Federal University of Pelotas, Pelotas 96055-630, Brazil; fabricioboscolo@gmail.com; 3 College of Physical Education, Federal University of Pará, Castanhal 68746-360, Brazil; vcoswig@gmail.com; 4 Department of Health, Exercise Science, and Recreation Management, Kevser Ermin Applied Physiology Laboratory, The University of Mississippi, Oxford, MS 38677-1848, USA; jploenne@olemiss.edu

**Keywords:** interval training, resistance training, body composition, aerobic training

## Abstract

Strategies aiming to promote weight loss usually include anything that results in an increase in energy expenditure (exercise) or a decrease in energy intake (diet). However, the probability of losing weight is low and the probability of sustained weight loss is even lower. Herein, we bring some questions and suggestions about the topic, with a focus on exercise interventions. Based on the current evidence, we should look at how metabolism changes in response to interventions instead of counting calories, so we can choose more efficient models that can account for the complexity of human organisms. In this regard, high-intensity training might be particularly interesting as a strategy to promote fat loss since it seems to promote many physiological changes that might favor long-term weight loss. However, it is important to recognize the controversy of the results regarding interval training (IT), which might be explained by the large variations in its application. For this reason, we have to be more judicious about how exercise is planned and performed and some factors, like supervision, might be important for the results. The intensity of exercise seems to modulate not only how many calories are expended after exercise, but also where they came from. Instead of only estimating the number of calories ingested and expended, it seems that we have to act positively in order to create an adequate environment for promoting healthy and sustainable weight loss.

## 1. Problem Statement

Although being overweight and/or obese are associated with numerous health risks, the prevalence of both are continuing to increase worldwide [1]. Obesity occurs when one’s energy expenditure is less than their energy intake, which creates an imbalance in energy. Other nutritional aspects, like the type of carbohydrates and fats as well as micronutrients, might also be considered [2]. If sustained, body fat will begin to accumulate. The treatment would include anything that results in an increase in energy expenditure (exercise) or a decrease in energy intake (diet). However, despite the short-term success of both exercise and diet, neither strategy seems to be effective for sustaining long-term changes in most individuals. The estimated weight loss for diet and/or exercise is approximately 2 kg by the end of two years in overweight and obese people [3,4]. Consequently, the probability of an obese individual attaining a normal weight is low and the probability of sustained weight loss is even lower. For example, Fildes et al. [5] estimated that the probability of a man with a body mass index between 40 and 44.9 kg × m^−2^ attaining a normal weight is only 1 in 1290, or 0.08%. Although this study did not control for exercise and diet, it provides important information about the difficulty in promoting sustainable weight loss. The objective of the present study is to present some reflections and invite the reader to critically analyze the strategies used for promoting weight loss. Herein, we highlight the importance of promoting the choice of exercise and supervision for those seeking or working with those seeking to sustain long-term changes in weight status/body fat.

## 2. Current Support for Exercise

In a recent article published by our group [6], 49 women were randomly assigned to perform two types of interval training (IT), high-intensity interval training (HIIT) and sprint interval training (SIT), and found positive changes in their adiposity measures (assessed by the sum of skinfolds) without changing their nutritional habits (assessed by 24-h dietary recalls). Here, we further explore if the results obtained were related to the nutritional changes. We tested if the participants that had higher decreases in caloric intake would show higher decreases in adiposity. To this purpose, we calculated the correlations between the changes in the nutritional and anthropometric variables using a bi-variated Pearson correlation model (Table 1). We also calculated regression models for the dependent variables (changes in anthropometric variables) using selected independent variables (changes in caloric ingestion). According to our results, there was no correlation between the changes in anthropometric measures and changes in caloric intake. Therefore, the changes in anthropometric measures could not be explained by nutritional changes.

By performing individual analyses, we noted that some participants increased their caloric intake and still achieved improvements in their anthropometric measures. In addition, there were some extreme cases like a participant that increased her caloric intake by more than 100% and decreased the sum of skinfold thickness by 20%. Another interesting example is a participant that increased her caloric intake by 35% and decreased the sum of skinfolds by 35%. On the other hand, another participant decreased her caloric intake by 24% and showed a slight increase of 1% in the sum of skinfolds.

We are aware that these analyses have some limitations such as the method used to assess caloric intake; however, the method is widely used and has been shown to be reproductible and previously validated [7,8,9,10]. We also do not have direct measures of the physical activity performed outside the training sessions; however, the participants were constantly asked about their physical activity habits throughout the experimental period to check if there were any relevant changes.

It is important to note that we were not the first group to describe decreases in the markers of adiposity or body composition in response to IT in the absence of caloric restriction [11,12,13,14]. Whilst some might find it intuitive that performing exercise would lead to fat loss due to the higher energy expenditure, previous studies showed that when energy intake was controlled, the addition of moderate-intensity exercise did not promote fat loss when compared with a control group, with [15,16,17] or without dietary interventions [16,18,19,20]. Even when there are significant changes, the magnitude of these changes is of limited biological significance [21,22]. The reason might be in the metabolic adaptations that occur in response to the interventions.

Some authors suggested that the metabolic changes that accompany a prolonged negative energy balance might be an important determinant of the ability to lose body fat [23]. In line with this, Reinhart et al. [24] reported that the success of dietary weight loss efforts is influenced by the energy expenditure response to caloric restriction. The authors classified some people as having a “thrifty” phenotype; that is, having large reductions in 24-h energy expenditure during fasting and smaller increases with overfeeding, while individuals with the opposite behavior were classified as “spendthrift”. According to the authors, greater decreases in energy expenditure during caloric restriction predict less weight loss, indicating the presence of thrifty and spendthrift phenotypes in obese humans. In agreement with this, Byrne et al. [25] suggested that, although lower-than-expected weight loss is often attributed to incomplete adherence to prescribed interventions, there are other factors that might influence the results, such as metabolic downregulation. In their study, they reported that a progressive metabolic adaptation in response to diet and exercise resulted in weight loss that was lower than predicted. Additionally, Fothergill et al. [26] also reported on metabolic adaptation when accompanying people that were submitted to an extreme weight loss program, and suggested that to obtain success in long-term weight loss, it is necessary to combat this metabolic adaptation so to avoid the counter-effects that mitigate the efforts to reduce body weight.

Regarding physical activity specifically, Pontzer [27] suggests that the current model (called additive or factorial) treats total energy expenditure simply as a product of body size and physical activity without considering the potential changes in energy allocation in response to the variations in activity levels. Therefore, the author proposes a model where energy expenditure adapts dynamically to the variations in physical activity to maintain total energy expenditure within some narrow physiological range.

In line with this, Westerterp et al. [28] investigated men and women that participated in a 40-week preparation for a half-marathon. The total energy expenditure and sleeping metabolic rate were measured at the 8th, 20th, and 40th weeks. According to the results, at the 20th and 40th weeks, total energy expenditure leveled off in both men and women, despite increasing exercise workloads and an increase in fat-free mass. The reductions in the sleeping metabolic rates suggest that metabolic adaptations occurred in response to the increased physical activity. Interestingly, previous studies found no increase in fat loss when aerobic moderate-intensity exercise was added to a diet, and reported that the groups that performed exercise showed a reduction in their resting metabolic rates [29,30].

So, instead of making people spend more calories through exercise, maybe we have to think on how to promote metabolic changes in order to overcome these physiological adaptations above-mentioned. In this case, not all exercises are equal.

In this regard, high-intensity training might be particularly interesting as a strategy to promote fat loss [31]. Irrespective to the number of calories spent during training, higher intensity exercise seems to promote many physiological changes that might favor long-term weight loss. For example, previous studies have shown that IT is able to promote the upregulation of important enzymes associated with glycolysis and beta-oxidation pathways [32,33,34,35], which occur to a greater extent than with moderate-intensity continuous exercise [34,35]. Interestingly, previous studies showed that some of these enzymes are under expressed in obese and ex-obese individuals [36,37], which might be related to energy expenditure and fat oxidation during resting [38,39]. Moreover, in the hours proceeding high-intensity exercise, there are noticeable increases in fat oxidation, which occurs either with IT [40,41,42,43] or resistance training [44,45,46,47,48]. Therefore, the intensity of exercise seems to modulate not only how many calories are expended after exercise, but also where they came from.

On the other hand, low to moderate continuous training has been shown to induce increases in fat synthesis after its cessation [49,50,51,52,53,54]. Whilst this does not mean that low- to moderate-intensity exercise will make people gain fat, this suggests that the metabolic adaptation to this form of exercise might, at least partially, compensate for the fat oxidized during exercise. This, summed with the reduction in nonphysical activity energy expenditure, might interfere with long-term fat loss.

Notwithstanding, long-term effects are obviously dependent on long-term adherence. In fact, this has been one of the main arguments to encourage low to moderate continuous training prescription and is based mainly on affective responses [55]. However, recent evidence showed that IT showed beneficial affective responses in both normal weight and overweight/obese people [56], which would be even better if the wide possibilities of IT were considered in training prescriptions to fit different people’s preferences [57]. Taken together, positive psychological and metabolic responses may explain long-term positive effects on unsupervised IT programs [58].

## 3. Considerations Moving Forward

So, the question is: could IT be the magic bullet for fat loss? To answer this, we conducted a systematic review and meta-analysis [31]. The results showed that IT promotes a greater reduction in absolute fat mass than moderate-intensity training, and SIT might be particularly interesting in that regard. However, it is important to recognize the controversy of the results regarding IT, which might be explained by the large variations in its application [59]. For this reason, we have to be more judicious about how exercise is planned and performed in order to guarantee that it will promote the necessary metabolic changes. Interestingly, in our article, supervision was a key factor, which might reinforce the argument that IT protocols need to be well-controlled. Moreover, it is important to observe that supervision is not usually provided in most studies involving other forms of exercise, which might also help to explain negative results. Based on the current evidence, it is our opinion that we must rethink the approaches used to promote fat loss. It is necessary to revise the mathematical model that pretends to fight overweight and obesity by simply increasing physical activity and/or decreasing caloric ingestion, since it can lead to frustrating results and induce unsustainable and ineffective behaviors. Based on the current evidence, we should look at how the metabolism changes in response to interventions instead of counting calories, so we can choose more efficient models that can account for the complexity of human organisms.

## 4. Conclusions

It is not our aim to present a final solution for fat loss, nor do we pretend to deny the importance of analyzing caloric expenditure. However, it seems that the human metabolism changes in response to what is done. Therefore, if we would like to calculate calories, it would be necessary to constantly evaluate our metabolic state in order to calculate our dietary needs, which is unfeasible. Therefore, instead of only estimating the number of calories ingested and expended, it seems that we have to act positively in order to create an adequate environment for promoting healthy and sustainable weight loss.

## Figures and Tables

**Table 1:** Correlations between changes in nutritional factors and changes in anthropometric measures.

Variables	Δ Energy Intake		Δ Carbohydrate Intake		Δ Protein Intake		Δ Lipid Intake	
	r	p	r	p	r	p	r	p
Δ body weight	0.06	0.7	0.19	0.18	−0.06	0.67	−0.01	0.97
Δ Body mass index	0.06	0.66	0.20	0.16	−0.06	0.69	−0.01	0.97
Δ waist circumference	0.17	0.25	0.17	0.24	0.21	0.14	0.22	0.14
Δ sum of ST	−0.20	0.17	−0.16	0.28	−0.05	0.73	−0.23	0.11
Δ triceps ST	−0.12	0.42	−0.06	0.71	−0.10	0.49	−0.11	0.45
Δ subescapular ST	−0.22	0.12	−0.22	0.14	−0.03	0.85	−0.21	0.15
Δ suprailiac ST	−0.13	0.36	−0.07	0.63	−0.01	0.97	−0.22	0.12
Δ abdominal ST	−0.06	0.70	−0.06	0.67	−0.03	0.87	−0.14	0.35
Δ thigh ST	−0.25	0.09	−0.24	0.10	−0.08	0.61	−0.19	0.18

*ST* = skinfold thickness; *r* = Pearson correlation coefficient, *p* = level of significance.
